# Subcutaneous Adipose Tissue-Secreted Proteins as Endocrine Regulators of Physical and Cognitive Function in Older Adults

**DOI:** 10.21203/rs.3.rs-6498803/v1

**Published:** 2025-06-03

**Authors:** Lauren Sparks, Cheehoon Ahn, Ian Tamburini, James Sanford, Mingqi Zhou, Farah Gamie, Reichelle Yeo, Carlos Viesi, Maria Pino, Katie Whytock, Lauren Oberlin, Theresa Mau, Joshua Adkins, Jamie Justice, Ashlee Wood, Zana Ross, Paul Piehowski, Chelsea Hutchinson Bunch, Kirk I. Erickson, Frederico Toledo, Nancy Lane, Peggy Cawthon, Anne Newman, Stephen Kritchevsky, Steven Cummings, Bret Goodpaster, Erin Kershaw, Marcus Seldin

**Affiliations:** Translational Research Institute, AdventHealth; Advent Health; University of California, Irvine; Pacific Northwest National Laboratory; University of California, Irvine; University of California, Irvine; Translational Research Institute, AdventHealth; University of California, Irvine; Translational Research Institute, AdventHealth; AdventHealth; California Pacific Medical Center; Pacific Northwest National Laboratory; XPRIZE Foundation; University of Pittsburgh; University of Pittsburgh; Pacific Northwest National Laboratory; Pacific Northwest National Laboratory; AdventHealth Research Institute, Neuroscience Institute, Orlando, FL, USA; Department of Medicine, Division of Endocrinology and Metabolism, University of Pittsburgh; University of Davis School of Medicine; Callifornia Pacific Medical Center Research Institute; University of Pittsburgh; Wake Forest School of Medicine; California Pacific Medical Center; University of Pittsburgh; University of California, Irvine

## Abstract

Declines in skeletal muscle and cognitive function in older adults have been linked to abnormalities in abdominal subcutaneous adipose tissue (ASAT), yet the underlying molecular mediators remain poorly understood. Here, leveraging ASAT transcriptomics and explant-conditioned media proteomics from participants in the Study of Muscle, Mobility and Aging (SOMMA; age ≥70 years, n = 229), we identified ASAT gene clusters and secreted proteins strongly associated with comprehensive assessments of physical and cognitive function in older adults. ASAT inflammation and secreted immunoglobulins were identified as key signatures of aging-associated physical and cognitive performance limitations. Systems genetics analysis confirmed secreted-SERPINF1 as a negative regulator of skeletal muscle contraction and highlighted its potential role in inducing inflammation in the heart *in silico*. Additionally, novel ASAT-secreted proteins such as NID2 and APOA4 were implicated in mediating ASAT crosstalk with skeletal muscle and brain *in silico*. Our framework provides insights into ASAT-driven tissue crosstalk underlying physical and cognitive performance in older adults and offers a valuable resource for understanding the role of ASAT in human aging.

## INTRODUCTION

Declining muscle mass, strength, gait speed, cardiorespiratory fitness, and cognitive function are typical with age and can lead to disability and death (1–3). While significant efforts have focused on uncovering the molecular drivers of aging-related changes within skeletal muscle (4, 5), brain (6, 7), and the muscle-brain axis (8, 9), growing evidence indicates that adipose tissue abnormalities may also contribute to aging-related functional declines (10, 11). Excessive mobilization of fatty acids and the secretion of pro-inflammatory factors from adipose tissue–common in aging adults–can lead to lipotoxicity and inflammation in skeletal muscle (12), contributing to metabolic impairments and functional declines in physical function (13). Chronic low-grade inflammation, largely attributed to adipose tissue, is associated with cognitive decline (14), and some adipose tissue-derived factors can directly regulate cognitive function (11). Senescent cells (cells that have reached irreversible cell fate but have not undergone programmed cell death) accumulate in adipose tissue during aging and are associated with physical and cognitive impairments (15, 16). Therefore, understanding the biology of adipose tissue in aging and identifying molecular mediators that link adipose tissue changes to physical and cognitive impairments can help develop novel strategies to address age-related functional declines.

Beyond inflammatory cytokines, adipose tissue secretes a variety of proteins, collectively referred to as ‘adipokines,’ which can exert significant biological effects on neighboring and distant organs (17). Visceral adipose tissue (VAT) has garnered much attention due to its tendency to increase with age and its higher protein secretory capacity per unit mass compared with subcutaneous adipose tissue (SAT) (18, 19); however, SAT likely serves as a major contributor to adipose tissue-mediated crosstalk because SAT is more susceptible to immune cell infiltrations and is the sentinel site of cellular senescence (20, 21). SAT also comprises the majority of total adipose tissue mass regardless of age (18, 22). Among SAT depots, the relative proportion of abdominal subcutaneous adipose tissue (ASAT) increases during aging (23), suggesting ASAT could be a significant contributor to adipose tissue-derived systemic effects. Therefore, profiling the proteins secreted by ASAT is a first step in understanding its direct contributions to physical and cognitive performance in aging.

The primary aim of this study was to characterize and interrogate the potential endocrine roles of ASAT-secreted proteins in physical and cognitive functions in older adults. To achieve this, we performed multi-omic profiling, including *in vivo* transcriptomics and *ex vivo* conditioned media (CM) proteomics in ASAT collected from participants in the Study of Muscle, Mobility and Aging – Adipose Tissue (SOMMA-AT) (24), who underwent comprehensive assessments of physical (i.e., walking and stair climbing speeds, leg power) and cognitive (i.e., executive function, and processing speed, memory, contrast sensitivity) performance. Using systems genetics analysis, we identified the expected and novel endocrine roles of selected secreted proteins in secondary target tissues associated with physical and cognitive function *in silico*. This workflow integrates ASAT-secreted protein profiling with the investigation of their systemic roles, providing a framework to better understand the link between aging-related adipose tissue abnormalities and physical and cognitive function in older adults thus enhancing the translational potential of clinical observations.

## RESULTS

### Subject characteristics

**Figure 1:**
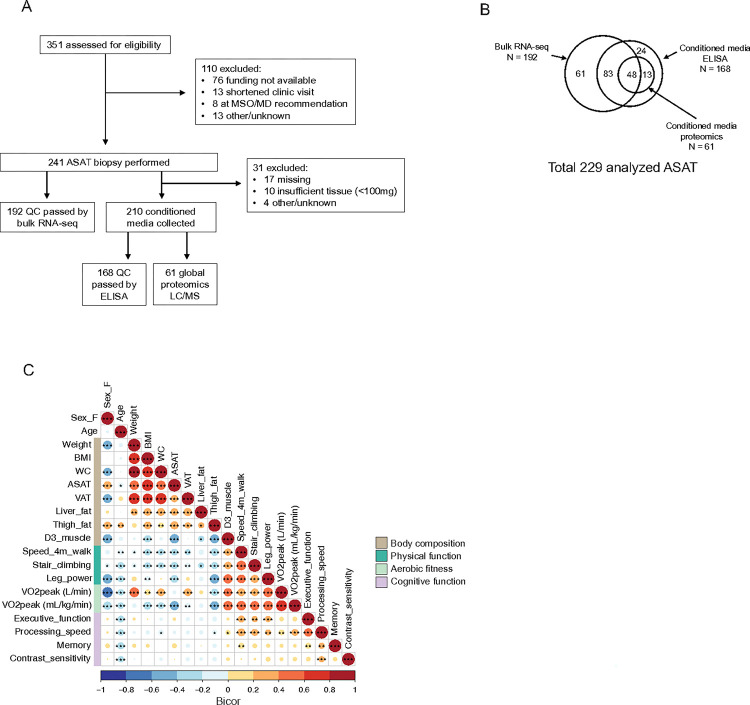
Study design and inter-clinical trait associations. A) Flow chart of subject recruitment, sample collection, and sample analysis. B) Venn diagram showing the distribution of sample analyses. C). Inter-clinical trait correlation of enrolled participants (N=229). MSO, Management service organization; MD, Medical doctor; ASAT, Abdominal subcutaneous adipose tissue; ELISA, Enzyme-Linked Immunosorbent Assay; LC/MS, Liquid Chromatography Mass Spectrometry. “adjusted p<0.05, “*adjusted p<0.01, “**adjusted p<0.001.

Out of the 351 participants who were assessed for eligibility in SOMMA-AT (24), 241 ASAT biopsy samples were acquired (Fig. 1A) and 229 samples were subsequently analyzed with at least one biochemical assay (i.e., bulk RNAseq, n = 192 or CM measurements (ELISA, n = 168; proteomics, n = 61)) (Fig. 1B). Participants were 76±4 years old and 52.4% (n = 120) were females (Table 1). Average BMI was 28.1±4.3 kg/m^2^, and participants with higher ASAT volume had higher ectopic fat mass (i.e., VAT, liver fat, thigh fat) and lower muscle mass and aerobic fitness (i.e., VO_2_peak) (Table 1). Significant intercorrelations among anthropometric and functional traits were identified (Fig. 1C). For example, adiposity measurements such as BMI, waist circumference (WC), ASAT volume, VAT volume, liver fat %, and thigh fat % exhibited high positive intercorrelations (adjusted p < 0.001) (Fig. 1C). Similarly, muscle mass, walking speed, stair climbing, leg power, and aerobic fitness were significantly positively intercorrelated (all adjusted p < 0.001), as were executive function, processing speed, and memory (all adjusted p < 0.01) (Fig. 1C). Contrast sensitivity, a visual processing measurement known to be tightly associated with cognitive abilities (25), was significantly correlated with processing speed (adjusted p < 0.001) (Fig. 1C). Moreover, physical functionalities were associated with executive function and processing speed (all adjusted p < 0.01) (Fig. 1C). Interestingly, among adiposity measurements, higher ASAT volume was strongly and consistently associated with lower muscle mass, walking speed, stair climbing, leg power, and aerobic fitness (ml/kg/min) (all adjusted p < 0.001) (Fig. 1C). Age was negatively correlated with walking speed, stair climbing, leg power, aerobic fitness, executive function, processing speed, memory, and contrast sensitivity (adjusted p < 0.01), consistent with the age-associated decline in physical and brain function (26, 27). Compared with males, females had greater ASAT volume and thigh fat % but less VAT and muscle mass (adjusted p < 0.05) (Fig. 1C).

### Association of ASAT transcripts with clinical traits

**Figure 2:**
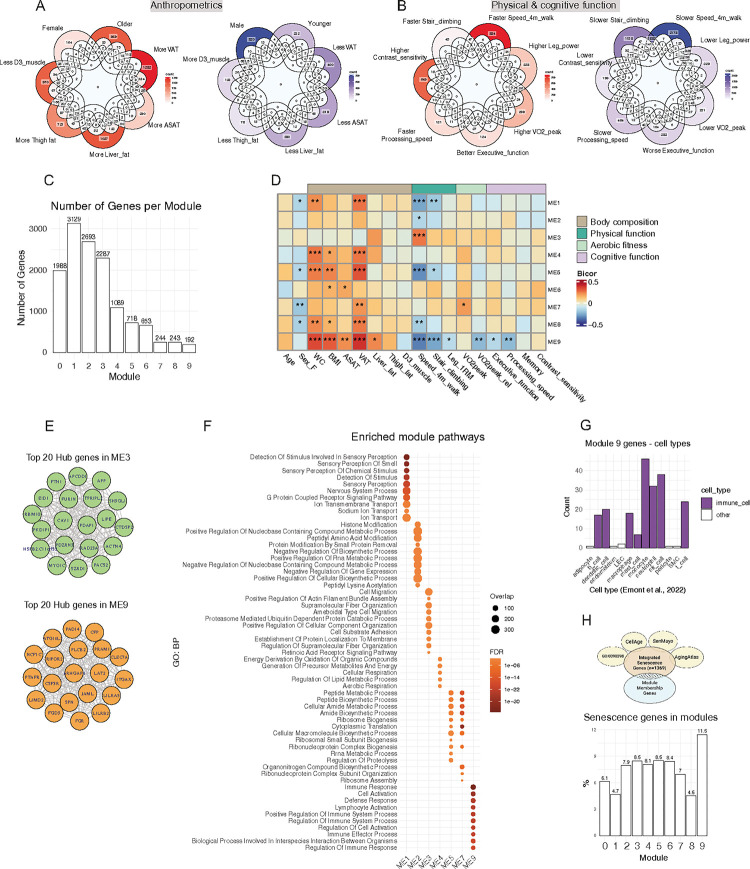
ASAT transcript clusters are associated with skeletal muscle and cognitive functions. A) Venn diagrams of significantly associated ASAT transcripts with anthropometric traits (p<0.05). B) Venn diagrams of significantly associated ASAT transcripts with functional traits (p<0.05). C) Number of genes in each module. Genes that do not fit well into any modules are placed in Module 0. D) Heatmap of correlation matrix between module eigengens and clinical traits. *p<0.05, **p<0.01, ***p<0.001. E) Network plots of the top 20 hub genes for modules 3 and 9. F) Top 10 overrepresented pathways from each module. There were no significantly enriched pathways from modules 6 or 8. G) Distribution of Module 9 member genes across different adipose tissue cell types. A total of 90 genes were mapped using cell-type marker genes from Emont et al., 2022. Genes were allowed to overlap across multiple cell types. H) An integrated senescence gene list (1369 genes) was curated by combining four different datasets. Then, the percentage of senescence genes per module was calculated. SkM, Skeletal Muscle; GO:BP, Gene Ontology: Biological Process; ME, Module eigengene; FDR, False Discovery Rate. Sample N = 192.

To investigate how ASAT biology is implicated in anthropometric and functional traits at the transcriptional level, we conducted bulk RNAseq on whole frozen ASAT samples (n = 192) and correlated gene expressions with clinical traits. Principal component analysis (PCA) of gene-trait correlations revealed that similar traits had similar associations with ASAT transcripts. For example, walking speed, stair climbing, muscle mass, and leg power - representing aspects of physical function - displayed similar associations (Figure S1). Likewise, cognitive function (e.g., executive function, processing speed, memory), and adiposity (e.g., ASAT, VAT, thigh fat) formed closely related clusters (Figure S1). However, despite their clustering, the degree and direction of these associations varied among similar traits. For instance, more transcripts were positively associated with VAT, liver fat, and thigh fat compared with transcripts negatively related to these fat depots (Fig. 2A). When comparing transcripts related to physical function, we found that only a small number of transcripts (n = 21) were intersected among faster walking speed, stair climbing, and stronger leg power (Fig. 2B), whereas 391 transcripts were correlated with poorer performances in these traits. These data suggest that ASAT transcripts may be informative about deteriorating physical function in older adults, but also some aspects of preserved physical function with age (Fig. 2B). Notably, the largest number of ASAT transcripts were associated with walking speed – a key functional outcome trait for mobility disability in SOMMA (28) (Fig. 2B, S2A). Overrepresentation analysis (ORA) from significantly associated transcripts with walking speed revealed upregulation of pathways related to protein quality control (e.g., ‘multivesicular body assembly’, ‘proteasome complex’, ‘response to unfolded protein’) and downregulation of ion transportation and detection of stimulus (Figure S2B). To further characterize gene networks associated with clinical traits, we conducted Weighted Gene Co-expression Network Analysis (WGCNA) and identified nine distinct modules (modules 1–9, see details in the Methods), each composed of co-expressed transcripts (Fig. 2C). Correlation between module eigengene (ME, the first principal component of the module representing overall transcript expression) and clinical traits revealed unique associations between transcript clusters and clinical traits (Fig. 2D). For example, module 3 was significantly associated with faster walking speed (Fig. 2D). Top 20 ‘Hub genes’ of module 3, which have high intramodular connectivity, included transcripts spanning multiple cellular functions such as cell growth (*ACTN4, PDAP1, APCDD1*) and substrate metabolism (*PACS2, FURIN, PDZRN3, LIPE, FTH*) (Fig. 2E). Aligning with this, ORA suggested enrichment in cell structural integrity, protein metabolism, apical junction, and p53 pathway (Fig. 2F, S2C). Conversely, module 9 was significantly associated with higher adiposity and poorer performance in walking speed, stair climbing, leg power, executive function, and processing speed (Fig. 2D). Top hub genes of module 9 were mostly involved in immune cell activation/signaling (e.g., *JAML, LAT2, RIPOR2, FGR, SPN, CFP, LILRA5, NCF1C, CLEC7A, PRAM1, LILRB2, PTAFR*) (Fig. 2E). Accordingly, enrichment analysis revealed substantial upregulation of immune response pathways (Fig. 2F, S2C), suggesting a strong connection between ASAT inflammation and poorer physical and cognitive performance in older adults. Cell-type analysis, using adipose tissue cell markers from Emont et al., 2022 (29), further supported these findings, showing that module 9 was predominantly composed of immune cell marker genes compared with non-immune cells (Fig. 2G). Given the established link between inflammation and cellular senescence (30), we also examined if module 9 membership genes may be associated with cellular senescence. An integrated list of senescence genes (n = 1369 genes) was curated by combining four different databases: CellAge (31), SenMayo (32), AgingAtlas (33), and GO:0090398 (cellular senescence). Module 9 showed the greatest overlap with the senescence genes, suggesting that cellular senescence may also have a role in poorer physical and cognitive functions (Fig. 2 H).

### Association of ASAT secreted proteins with clinical traits – Adiponectin and Leptin

**Figure 3:**
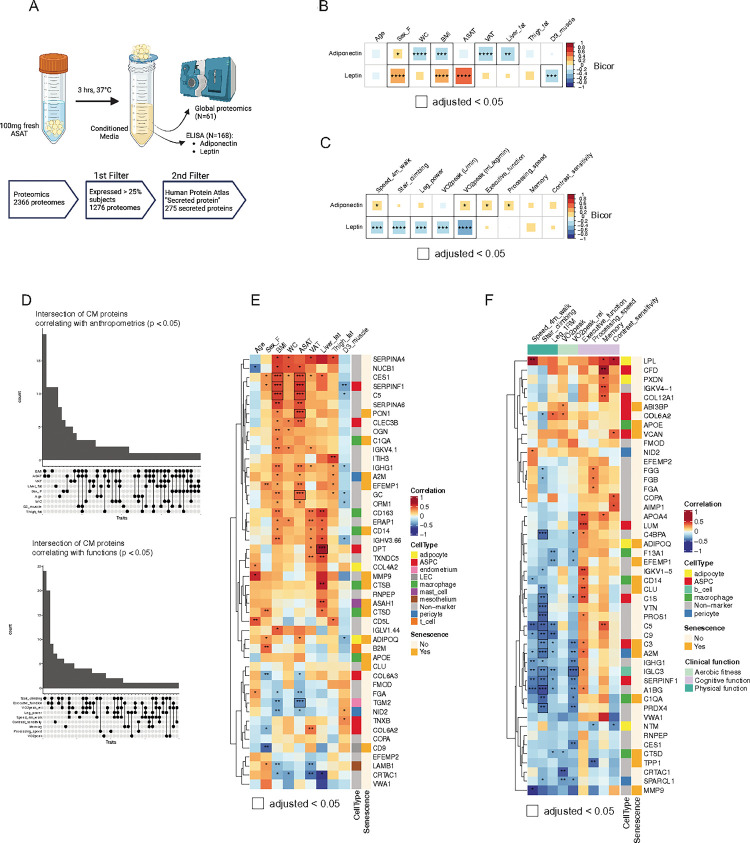
ASAT-secreted proteins are associated with skeletal muscle and cognitive functions. A) Schematics of ASAT CM collection and secretome measurements. Correlation matrix between ELISA-measured adipokines (adiponectin and leptin) and B) anthropometric and C) functional traits. Sample N = 168. D) Upset plots depicting intersections of anthropometric/functional traits on significantly associated secreted proteins measured by proteomics (p<0.05). Bars corresponding to black dots represent the count of significantly associated proteins. Connecting lines represent the intersection of significantly associated proteins among traits. Sample N=61. Correlation matrix between proteomics-measured secreted proteins and E) anthropometric or F) functional traits. ‘Non-marker’ represents transcripts that were not identified as cell-type specific markers (i.e., |log⁡2FC|<1). Top 5 secreted proteins are shown for each trait if there were more than 5 significantly associated secreted proteins. * p<0.05, ** p<0.01, *** p<0.001. **** p<0.0001. Black outline indicates adjusted p<0.05, which was obtained from Benjamini-Hochberg multiple testing correction. Sample N=61. CM, Conditioned media; Bicor, Biweight midcorrelation coefficient.

To characterize ASAT-secreted protein profiles from older adults, we measured the abundance of well-known adipokines – adiponectin and leptin – via targeted immunoassay (ELISA) from ASAT explant conditioned media (CM) in a subset of participants (n = 168) (Fig. 3A). Adiponectin is typically inversely associated with adiposity and has insulin-sensitizing and anti-inflammatory effects on tissues such as the liver and muscle (34, 35). In contrast, leptin regulates satiety, is strongly correlated with adiposity, and its excess is often associated with adverse cardiometabolic health outcomes (36). The abundance of secreted adiponectin was negatively correlated with adiposity measures (i.e., WC and BMI) and ectopic fat (i.e., VAT and liver fat) and positively correlated with walking speed and executive function (all adjusted p < 0.05) (Fig. 3B, C). In contrast, the abundance of secreted leptin was greater in females and was associated with higher ASAT volume and lower muscle mass (Fig. 3B). Leptin was also strongly correlated with worse walking speed, stair climbing, leg power, and aerobic fitness (all adjusted p < 0.05) (Fig. 3C).

### Association of ASAT secreted proteins with clinical traits – Proteomics

To more globally understand the impact of ASAT-secreted proteins on clinical traits, we next conducted untargeted global proteomics on ASAT-CM in 61 participants (Fig. 3A). A total of 2366 proteins were identified, with 1276 proteins expressed in at least 25% of the samples (Fig. 3A). Secreted proteins were annotated using the secreted protein database from The Human Protein Atlas (Supporting information 1), resulting in the retention of 275 ‘secreted proteins’ (Fig. 3A). Final secreted proteins were also mapped for cell types and senescence using the marker gene lists used in Fig. 2.

BMI and ASAT volume were the top 2 anthropometric traits that were mostly associated with the abundance of secreted proteins, intersecting with other traits (Fig. 3D), suggesting a tight link between adiposity and ASAT-secreted proteins. For example, the secreted abundance of SERPINF1 (also known as pigment epithelium-derived factor (PEDF)), a marker for adipose stem and progenitor cells (ASPC) and known for its inhibitory effect on angiogenesis (37), was associated with higher BMI and ASAT volume (adjusted p < 0.05) (Fig. 3E). Surprisingly, some secreted proteins more well-known to be produced from the liver, such as PON1, a high-density lipoprotein (HDL)-associated enzyme that contributes to anti-inflammatory effects in endothelial cells (38), and GC, a vitamin D-binding protein, were detected from ASAT-CM, and their abundance was also significantly correlated with higher ASAT volume (Fig. 3E). The abundance of secreted DPT, also a marker for ASPC and an adipokine that can induce fibrosis and inflammation in the liver and VAT (39, 40), was strongly associated with higher liver fat (bicor = 0.79, adjusted p < 0.05) and also with VAT (p = 0.019) (Fig. 3E). Many of the secreted proteins associated with anthropometric traits were markers for ASPCs and macrophages (Fig. 3E).

We then examined how ASAT-secreted proteins may relate to functional traits in older adults. Poorer stair climbing was associated with numerous secreted proteins, including complement proteins (e.g., C5, C9, and C4BP4), SERPINF1, A2M, A1BG, CLU, VTN, and PROS1 (all adjusted p < 0.05) (Fig. 3D, F). Many of these secreted proteins were also negatively associated with walking speed and leg power (p < 0.05) (Fig. 3F), suggesting a link between these secreted proteins and an overall decline in physical function. For instance, VTN, a multifunctional glycoprotein involved in cell adhesion and tissue remodeling, has reduced gene expression in murine skeletal muscle during post-injury regeneration (41). Conversely, increased VTN expression has been observed in bioengineered human skeletal muscle models for muscular dystrophy (42), supporting the inverse relationship between VTN and physical function. In addition to the positive correlation with walking speed, lipoprotein lipase (LPL) was associated with better memory performance and contrast sensitivity (both p < 0.05) (Fig. 3F), aligning with previous findings that linked circulating (43,44) and brain-specific (45) LPL expression with cognitive function. Among the secreted proteins that were positively associated with executive function (p < 0.05, Fig. 3F), APOA4, LUM, and CLU are reported to be expressed in the brain (46, 47), and lower circulating abundance of APOA4 and CLU in circulation has been observed in individuals with mild cognitive impairment and Alzheimer’s Disease (48, 49), collectively suggesting that these secreted proteins may contribute to better cognitive functions. Despite tight inter-correlation between physical and cognitive functions (Fig. 1C), some secreted proteins were associated with reduced physical function but better executive function (e.g., C1S, CLU, A1BG) or memory (e.g., C5), indicating a possibility of the multifaceted role of ASAT-secreted proteins (Fig. 3F). Together, our characterization of ASAT-secreted proteins derived from *ex vivo* conditioned media provides new insights into the endocrine role of ASAT that may impact physical and cognitive function in older adults.

### Integration of WGCNA and secreted proteins reveals immunoglobulins as a signature of aging-related functional declines.

**Figure 4:**
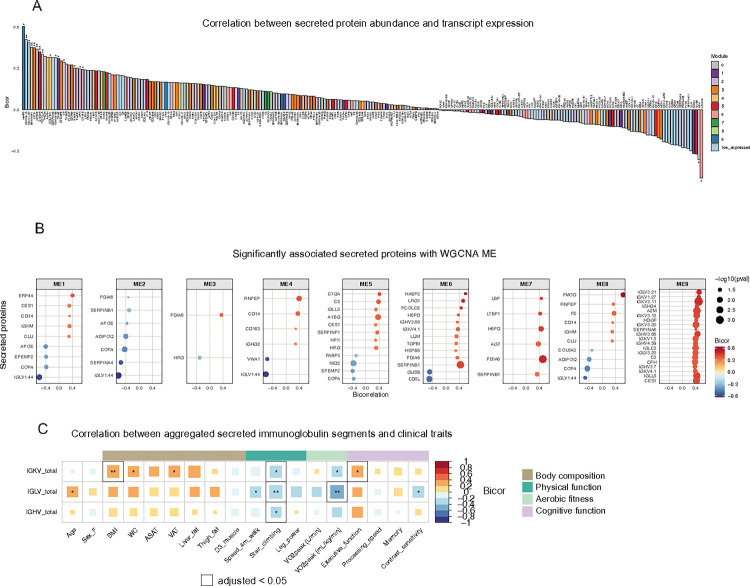
Integration of ASAT transcripts and secreted proteins. A) Correlation between secreted protein abundances and their protein-coding transcript expressions. ‘Low expressed’ refers to genes that were excluded before WGCNA analysis due to lower expression. *p<0.05, **p<0.01. B) Secreted proteins that are significantly associated with module eigengenes. (p<0.05). Top 20 secreted proteins are shown. C) Correlation matrix between mean abundance of immunoglobulin chains and clinical traits. *p<0.05, **p<0.01. The black outline indicates adjusted p <0.05, which was obtained from Benjamini-Hochberg multiple testing correction. Sample N=48.

To explore the relationship between ASAT transcripts and secreted proteins, we correlated the expression of 254 transcripts (matching genes for secreted proteins) with the abundance of secreted proteins and overlaid their module memberships (Fig. 4A, S3A). MMP9, a member gene for module 9 and mapped as a marker for cellular senescence from the curated senescence list (Fig. 3E), exhibited the highest correlation (bicor = 0.6,p < 0.05) between transcript expression and protein abundance (Fig. 4A). The abundance of secreted MMP9 was associated with age and slower walking speed (Fig. 3E, F). We also found several other significant associations between ASAT transcript expression and secreted protein abundance, such as SERPINF1, IGKV1–5, and EFEMP2 (all p < 0.05, Fig. 4A). The associations between transcript expression and secreted protein abundance of HPX and LAMA5 exhibited significant inverse relationships, implying complex post-transcriptional/translational modifications (both p < 0.05, Fig. 4A). We further analyzed correlations between the abundance of secreted proteins and MEs to understand relationships between gene clusters and protein secretion. Notably, the secreted abundance of numerous variable regions of immunoglobulins (e.g., IGLVs, IGKVs, IGHVs), primarily produced by plasmacytes, were significantly associated with ME9 (Fig. 4B), suggesting that excessive immunoglobulin production from ASAT may be tightly linked to poorer physical and cognitive functions. The overall correlation trends were similar across aggregated variable regions (IGKV, IGLV, and IGHV); however, differences in significance were observed (Fig. 4C). Notably, IGKV abundance was significantly associated with higher adiposity and better executive function (Fig. 4C). While all variable regions correlated with poorer stair climbing, IGLV abundance was also significantly correlated with slower walking speed, lower aerobic fitness, and poorer contrast sensitivity (Fig. 4C). Correlation analysis of aggregated IGLV abundance and ASAT transcripts revealed both positive and negative associations (p < 0.05) (Figure S3B), with enriched pathways in positively associated genes indicating immune/inflammatory upregulation, while pathways in negatively associated genes suggested organic acid and lipid metabolic processes (Figure S3C).

### Systems genetics analysis suggests potential ASAT-mediated endocrine effects on multiple tissues

**Figure 5:**
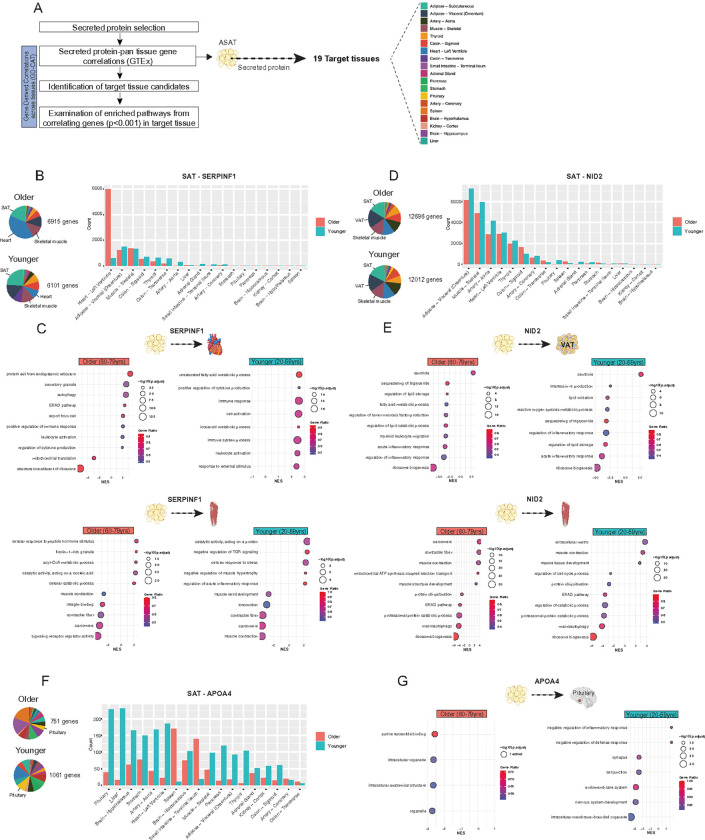
Validation of endocrine effects of ASAT-secreted proteins via systems genetics. A) Workflow of GD-CAT analysis. B) Pie chart and bar plots show significantly associated genes (adjusted p<0.001) in all tissues with ASAT-derived protein candidates using GTEx cohorts (“Younger”, 20–59 years; “Older”, 60–79 years). Enrichment plot shows overrepresented pathways in target tissues from genes significantly associated with ASAT-derived protein candidates using GTEx cohorts. B) Pan tissue correlation with SAT-SERPINF1. C) Enrichment plots from target tissue genes (heart, skeletal muscle) that correlated with SAT-SERPINF1. D) Pan tissue correlation with SAT-NID2. D) Enrichment plots from target tissue genes (VAT, skeletal muscle) that correlated with SAT-NID2. F) Pan tissue correlation with SAT-APOA4. C) Enrichment plots from target tissue genes (pituitary) that correlated with SAT-APOA4. Gene ontology databases (Biological process, BP; cellular component, CC; molecular function, MF) were used for enrichment analysis. GD-CAT, Gene-Derived Correlations Across Tissues; GTEx, The Genotype-Tissue Expression; SAT, Subcutaneous Adipose Tissue; VAT, Visceral Adipose Tissue; NES, Normalized Enrichment Score.

Discovering inter-organ signaling networks through gene-gene correlations across tissues has become increasingly useful in tissue crosstalk research due to its robustness and versatility (50–54). Gene-derived correlations across tissues (GD-CAT) (55) is a platform we developed that leverages transcriptome data from 135 “Older”(age:60–79) and 175 “Younger”(age:20–59) participants in the post-mortem GTEx cohorts (56) to identify candidates of target tissues for a given gene of origin tissue and further examine enriched biological pathways in the target tissues that may explain the action of crosstalk (Fig. 5A). Leveraging the relationship between ASAT transcripts/secreted proteins and our clinical traits, we highlight the potential endocrine effects of selected ASAT-secreted proteins on secondary target tissues *in silico*.

*SERPINF1* is linked to adiposity and adverse metabolic health (57, 58). *SERPINF1* transcript expression strongly correlated with its secreted protein abundance (Fig. 4A), and SERPINF1 protein abundance was negatively associated with walking speed, stair climbing, leg power, and aerobic fitness (Fig. 3F). Additionally, by using BioGPS, a panel that provides multi-tissue expression of genes (59, 60), we found that SAT is a primary secretory tissue for *SERPINF1* (Figure S4). In the Older cohort, SAT-*SERPINF1* correlated most strongly with genes in the heart and skeletal muscle, while its correlations were weaker in the Younger cohort (Fig. 5B). Enrichment analysis of correlating genes in the heart with SAT-*SERPINF1* suggested upregulated cytokine production and inflammatory responses in both Older and Younger cohorts, while mitochondrial translation was downregulated only in the Older cohort (Fig. 5C). Moreover, enrichment analysis of genes in skeletal muscle correlating with SAT-*SERPINF1* revealed a pronounced downregulation of muscle contractility components, accompanied by upregulation of inflammatory pathways and catabolic processes in both age groups (Fig. 5C).

We also inspected *NID2*, which encodes cell adhesion protein Nidogen2, given its positive associations with muscle mass and walking speed (Fig. 3E, F). Secreted abundance of NID2 was also negatively associated with module 5 (Fig. 4B), corroborating the positive relationship between NID2 and physical function (Fig. 2D). Abundantly expressed in SAT (Figure S5), GD-CAT analysis identified VAT and skeletal muscle as the top secondary tissues associated with SAT-*NID2* in both age groups (Fig. 5D). Enrichment analysis of genes in VAT correlating with SAT-*NID2* revealed substantial downregulation of inflammatory responses, lipid metabolism, and ribosome biogenesis (Fig. 5E). In skeletal muscle, SAT-*NID2* was associated with enhanced muscle contraction- and component-related terms, while protein catabolism, ER stress, autophagy, and ribosome biogenesis were downregulated (Fig. 5E).

Although SAT is not a primary origin for APOA4 (Figure S5), the secreted abundance of APOA4 displayed positive associations with executive function and memory performance (Fig. 3F), suggesting a link between ASAT and cognitive function. GD-CAT revealed distinct secondary tissue gene correlations between Older vs. Younger cohorts, where more than 5-fold genes were correlated with SAT-APOA4 in the pituitary and hypothalamus in the Younger cohort compared with the Older cohort (Fig. 5F). In the Younger cohort, enriched pathways from pituitary genes that were associated with SAT-APOA4 included upregulation of anti-inflammatory responses. Conversely, pathways related to organelle structure were downregulated in both Younger and Older cohorts (Fig. 5G).

### ASAT transcript/secreted protein profile in older adults with high physical fitness

**Figure 6:**
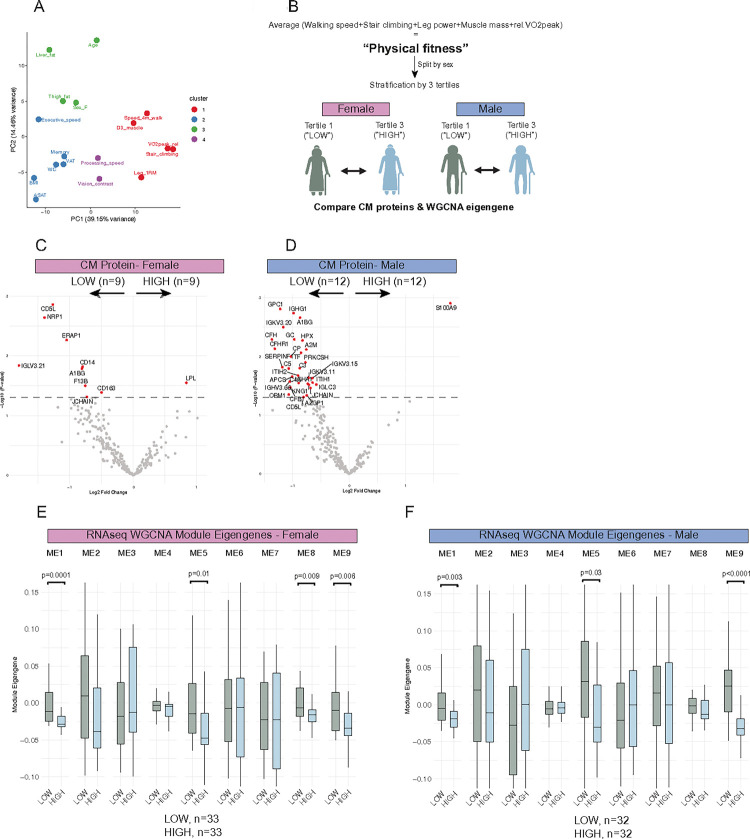
Older adults with higher ‘physical fitness’ have distinct ASAT transcripts and secreted protein profiles. A) PCA analysis of the correlation between clinical traits and secreted proteins. K-means clustering was used to group clinical traits into clusters. The number of centers was set to four. Sample N=61. B) Flow of participants stratification by ‘physical fitness’. C) Volcano plot of differentially expressed secreted proteins in LOW vs. HIGH females (p<0.05). LOW, n = 9; HIGH, n=9. D) Volcano plot of differentially expressed secreted proteins in LOW vs. HIGH males (p<0.05). LOW, n=12; HIGH, n=12. E) Comparison of WGCNA ME between LOW vs. HIGH females. LOW, n=33; HIGH, n=33. F) Comparison of WGCNA ME between LOW vs. HIGH males. LOW, n=32; HIGH, n=32. Rel.VO_2_peak, Relative VO_2_peak (ml/kg/min). PCA, Principal component analysis; ME, Module eigengene.

Given the associations of ASAT-secreted proteins with clinical traits, we explored how these secreted proteins and transcript profiles may manifest in participants with distinct functionalities. Using k-means clustering (k = 4) based on PCA of clinical trait associations with secreted proteins, we confirmed that walking speed, stair climbing, leg power, relative VO_2_peak (ml/kg/min), and muscle mass clustered together (Fig. 6A), which aligned very closely with PCA results of clinical trait associations with *in vivo* transcripts (Figure S1). We calculated the average z-scores for these five traits as a composite measure of ‘physical fitness’ and stratified participants into tertiles, in each sex. The first tertile (‘LOW’) and the third tertile (‘HIGH’) were compared for secreted proteins and transcript modules (Fig. 6B). By design, the five physical fitness component traits were significantly lower in both LOW men and women compared to their HIGH counterparts (Table 2). In addition, although participants were stratified by physical fitness measures, the HIGH group exhibited lower adiposity and higher cognitive function (Table 2). Between HIGH and LOW females (n = 9 each), 10 secreted proteins were differentially expressed: 1 upregulated and 9 downregulated in the HIGH group (p < 0.05) (Fig. 6C). Many of the downregulated secreted proteins in HIGH females were immune cell-related proteins (e.g., IGLV3–21, CD163, CD14, CD5L, ERAP1), suggesting suppressed immune activity in ASAT of older females with high physical fitness, low adiposity, and high cognitive function. Conversely, secreted LPL was more abundant in HIGH females compared with LOW females (p < 0.05) (Fig. 6C). In HIGH vs. LOW men (n = 12 each), 30 out of 31 differentially expressed secreted proteins were downregulated, including complement proteins (C3, C5, C4a, CFH), immunoglobulin variables (IGHG1, IGKV3–20, IGKV3–15, IGKV3–11, IGHV3–66, IGLC3), and iron carrier proteins (HPX, TF) (Fig. 6D). The abundance of secreted LPL and S100A9 was upregulated in HIGH in females and males, respectively (p < 0.05) (Fig. 6D). We also compared ME between LOW and HIGH females (n = 32 each) and males (n = 33 each) (Fig. 6E, F). For both females and males, ME for modules 1 (enriched for ion transport; Fig. 2F), 5 (enriched for ribosome biogenesis and amide metabolism; Fig. 2F), and 9 (enriched for inflammation; Fig. 2F) were lower in HIGH vs. LOW (all p < 0.05) (Fig. 6E, F). ME for module 8 was lower in HIGH females (p < 0.01) (Fig. 6E).

## DISCUSSION

Our multi-omic analysis, integrating *in vivo* RNAseq and *ex vivo* conditioned media (CM) proteomics of ASAT collected from older adults, combined with detailed measurements of physical and cognitive performance, provided a comprehensive characterization of ASAT transcripts and secreted proteins that may contribute to functional health. A key finding was the novel association of ASAT-secreted proteins – including SERPINF1, NID2, and APOA4 – with physical and cognitive functions in older adults. We also identified a distinct gene cluster, primarily immune cell-derived, that was strongly associated with poor physical and cognitive function. Integration of transcriptomics and CM proteomics highlighted potential aging-related molecular signatures, such as MMP9 and immunoglobulins, linked to functional decline. Our study demonstrates the utility of systems genetics analysis in examining the endocrine roles of ASAT-secreted proteins in secondary tissues *in silico*. Stratification of older adults by physical fitness levels enabled further insights into ASAT gene expression and secreted protein profiles associated with physical fitness, as well as cognitive function. This unique approach, integrating ASAT transcript expression, secreted protein characterization, and estimation of ASAT endocrine roles, advances our understanding of ASAT that may impact physical performance and cognitive function in aging populations.

Our approach to the relatively short-term (i.e., 3-hour) ASAT ‘organ culture’, which preserves intact ASAT fragments in a complete culture medium (61), captured a pool of secreted proteins that reflect the *in vivo* transcriptomic profile of ASAT. The observed associations of secreted adiponectin and leptin abundances with adiposity align with prior literature (62–65), supporting the reliability of our CM experimental model. Interestingly, the positive correlation of secreted adiponectin abundance with walking speed and executive function contrasts with the ‘adiponectin paradox’, where higher circulating adiponectin is linked to frailty and poor physical and cognitive performances in older adults (66–68). This discrepancy suggests that the ‘secretory capacity’ of ASAT for adiponectin per se may not underlie the negative association between circulating adiponectin and health outcomes in aged populations While leptin promotes skeletal muscle growth and function during development (69), our findings align with studies indicating its association with reduced skeletal muscle function in older populations (70, 71), possibly due to aging-specific mechanisms. For example, leptin has been shown to drive IL6-mediated inflammation in the skeletal muscle of aged mice (72), indicating its complex and context-dependent role in aging-related skeletal muscle health.

In this study, we aimed to identify ASAT-secreted proteins associated with physical and cognitive function and investigate the potential endocrine roles of secreted proteins in secondary tissues using GD-CAT. PEDF (encoded by *SERPINF1*), a known adipokine, has been implicated in the pathophysiology of type 2 diabetes due to its strong association with cardiometabolic risk factors such as obesity-associated inflammation and insulin resistance (73, 74). Treatment of human-derived skeletal muscle cells with PEDF inhibits Akt phosphorylation and activates NF0κB and p38 MAPK pathways, inducing insulin resistance and inflammation (75). Our finding that the protein abundance of secreted SERPINF1 was associated with higher adiposity and reduced muscle mass, walking speed, stair climbing, and leg power supports this link, as insulin resistance and elevated inflammation are often associated with aging, sarcopenic skeletal muscle (10). Additionally, the adipose *SERPINF1* and skeletal muscle gene correlation observed in GD-CAT analysis – showing suppressed muscle contraction and enhanced inflammatory responses – aligns with existing literature and highlights the utility of *in silico* systems genetics analysis in studying tissue crosstalk. Interestingly, our finding suggests that the heart could be another major target tissue of ASAT-derived SERPINF1, particularly in older adults, where it may interfere with mitochondrial protein translation while inciting inflammatory responses. Conversely, nidogen-2 (encoded by *NID2*) was associated with faster walking speed and negatively correlated with module 5, a cluster linked to higher adiposity and slower walking and stair climbing speeds. GD-CAT analysis further supported this, showing upregulation of muscle contraction and development in skeletal muscle genes correlated with adipose *NID2*, suggesting its potential role in promoting favorable adaptations in aging muscle. Our finding also suggests adipose nidogen-2 as a negative regulator of VAT inflammation and lipid uptake, indicating a favorable role of ASAT-secreted nidogen-2 in cardiometabolic health. Although circulating apolipoprotein A4 (encoded by *APOA4*) is largely produced by the intestine (76), the positive associations between ASAT-secreted apolipoprotein A4 abundance and memory and executive function suggest ASAT-secreted apolipoprotein A4 may play a role in adipose-brain crosstalk. This relationship may weaken in older adults, as indicated by GD-CAT analysis. In addition to aiding lipid metabolism, apolipoprotein A4 possesses anti-inflammatory and antioxidant properties (77, 78), which are processes known to support cognitive health. Moreover, deficiency of APOA4 promotes β-amyloid formation and results in cognitive deficits, suggesting a higher abundance of apolipoprotein A4 may be favored for preserving cognitive functions.

Our findings reveal a strong correlation between module 9, a unique cluster of immune cell-derived transcripts, and reduced physical and cognitive functions, supporting the role of ASAT inflammation and senescence in age-related functional impairments (79, 80). Proinflammatory and senescence-associated secretory phenotype (SASP) cytokines, such as TNF, are known to inhibit myogenesis and induce insulin resistance in skeletal muscle (81, 82). Peripheral immune processes, including chronic pro-inflammatory signaling, have also been implicated in cognitive decline, neurodegeneration, and dementia-related pathology, though the specific pathways are not yet understood. Using plasma proteomics, recent studies have revealed multiple immune-related proteins associated with brain aging and increased dementia risk (83, 84). Our findings extend this work by identifying ASAT as a potent source of immunologically relevant proteins that may contribute to cognitive decline in older adulthood. Additionally, recent reports suggested immunoglobulin-associated senescence as a hallmark of aging, with accumulation of immunoglobulin G (IgG) observed in adipose tissue (85) and multiple tissues (86) in aged mice. Integrating WGCNA and CM proteomics, we identified ASAT-secreted immunoglobulins were strongly associated with reduced walking speed, stair climbing, aerobic fitness, and contrast sensitivity, suggesting they could be additional signatures of aging-associated ASAT abnormalities that may contribute to functional declines in older adults. While immunoglobulins are primarily released by plasmacytes, other cells may also contribute (86). The exact role of ASAT-specific immunoglobulin accumulation in driving inflammation and senescence remains unclear, though increased immunoglobulin release may prime ASAT macrophages to secrete proinflammatory cytokine or systemically accelerate cellular senescence (85–87). Our findings suggest that the biological role of immunoglobulins may vary by gene segment. For example, while many secreted immunoglobulin gene segments were positively associated with module 9, IGLV1–44 abundance was negatively associated with modules 1, 2, 4, and 8, suggesting a linkage with lower adiposity and faster walking speed. ASAT-secreted MMP9, a gelatinase involved in extracellular matrix protein turnover, also emerged as a signature of aging due to its strong correlation between transcript expression and secreted protein abundance, as well as its association with age. While MMP9 can promote collagen degradation and angiogenesis, counteracting fibrosis (88), elevated MMP9 levels in ASAT or plasma have been linked to insulin resistance and cardiovascular disease risk (89, 90). Lifestyle interventions, such as exercise or weight loss, have been shown to reduce MMP9 protein abundance or gene expression in ASAT – at least in young or middle-aged adults – indicating that excessive ASAT MMP9 may be unfavorable (91,92). Reduced enzymatic activity of MMP9 could underlie the overproduction of MMP9 (93), yet the exact mechanism remains unclear.

Our exploratory analysis comparing participants stratified by the composite physical fitness score revealed ASAT characteristics that may be particularly relevant to older adults with reduced physical function. In both female and male tertile comparisons, lower physical fitness was associated with a higher abundance of inflammatory factors and immunoglobulin segment, including JCHAIN – a polypeptide that facilitates IgA and IgM polymer formation and exocrine secretion (94) – suggestive of ASAT as a source of excessive secretion of various immunoglobulin classes in older adults with poor physical fitness. Conversely, older adults with higher physical fitness exhibited elevated abundance of LPL and S100A9 (a neutrophil marker gene that encodes calprotectin) in females and males, respectively. S100A9 has been shown to enhance insulin-independent glucose uptake in skeletal muscle via TLR4 and attenuate systemic inflammation (95), potentially benefiting muscle function and mobility. While stratification was based on a composite physical fitness score, differences in executive function and processing speed between tertiles suggest that the identified secreted proteins and transcripts could also be linked to cognitive function.

While our study design enabled comprehensive profiling of ASAT-secreted proteins associated with physical and cognitive functions in older adults, it is important to acknowledge some of the limitations inherent to observational studies. Although our findings of ASAT-secreted proteins that were significantly associated with physical or cognitive function are intriguing, the direct causal link remains unclear, and the definitive mechanistic validation requires further exploration. However, *in silico* GD-CAT analysis provided insight into the expected endocrine roles of secreted proteins, which aligned well with established literature (e.g., *SERPINF1*), lending credibility to our findings. Also, it is possible that our relatively short incubation of ASAT explants (i.e., 3 hours) may not have fully captured the secreted proteins in the conditioned media. While we acknowledge that incubation time may differentially impact protein secretion, our approach enabled integrative analysis of transcripts and secreted proteins, which led to the identification of secreted immunoglobulins as potential mediators of ASAT-driven physical and cognitive function impairments and aligned with established literature of classic adipokine secretion of leptin and adiponectin associations with adiposity and other clinical outcomes. Our exclusion criteria (e.g., inability to complete a 400 m walk in 15min, BMI>40 kg/m^2^) may limit broader applicability, as ASAT characteristics could differ in populations with very slow walking speed or severe obesity, both strong predictors of cardiovascular mortality (96, 97). These criteria were selected for our study to enable participants to complete detailed mobility and body composition assessments, which strengthened the observed correlations between clinical traits and ASAT-secreted proteins. Future studies should include higher-risk populations to expand the scope of tissue crosstalk research.

In summary, our findings indicate that transcriptional and secretome alterations in ASAT are highly associated with, or even underlie, physical and cognitive performance in older adults. Notably, ASAT inflammation and immunoglobulin production may be key factors linked to reduced physical and cognitive functions. Using global proteomics from ASAT-conditioned media, we identified numerous secreted proteins with significant associations with clinical functionalities. Furthermore, we present a workflow that integrates multi-omics analyses with systems genetics analysis, offering a robust approach to enhance the translatability of clinical insights in tissue crosstalk research. Overall, our study underscores the central role of ASAT in regulating physical and cognitive functions in aging adults, providing a valuable platform for future investigations into tissue crosstalk.

## METHODS

### Participant recruitment

A total of 241 participants aged 70 and older were recruited as part of the SOMMA (https://www.sommastudy.com/) from April 2019 to December 2021 across two clinical sites; the University of Pittsburgh and Wake Forest University School of Medicine (24). Detailed descriptions of the parent SOMMA study cohorts and clinical measurements are available here (28). Individuals who reported being unable to walk ¼ mile or climb a flight of stairs, had an active malignancy, suffered from advanced chronic conditions (e.g., severe heart or lung disease preventing them from walking ¼ mile, severe kidney disease requiring dialysis, Parkinson’s disease, or dementia), or had body mass index (BMI) ≥ 40kg/m^2^ were excluded from the study. Additionally, individuals with medical contraindications to biopsy or MRI, such as chronic anticoagulation or incompatible metal implants, were also excluded. In-person assessments at enrollment ensured that all participants were capable of walking 400 meters. All participants provided written informed consent, and the study was approved by the Western Institutional Review Board (20180764) for all participating sites.

### Body size and composition measurements

The SOMMA baseline evaluations were conducted over several days and included self-reported information on sex, race, ethnicity, health history, medication use, diet, and physical activity. Height was measured using stadiometers, and weight was recorded on digital scales. VO2 peak was assessed via cardiopulmonary exercise testing (CPET) (98). Body composition was measured using magnetic resonance imaging (MRI). Whole-body MR scans were processed with Dixon water-fat imaging using AMRA Researcher (AMRA Medical AB, Linköping, Sweden) to estimate the volumes of abdominal subcutaneous adipose tissue (ASAT), visceral adipose tissue (VAT), thigh fat infiltration, and liver fat percentage (%). Total skeletal muscle mass (D_3_C muscle) was assessed by validated D_3_C dilution method (99).

### Fitness and physical performance

#### Walking speed.

4m walking speed was measured during the Short Physical Performance Battery (SPPB), which is a well-established tool in clinical research for evaluating lower extremity function. Walking speed (m/sec) was assessed over a 4-meter walking course.

#### Stair climbing.

Participants climbed up and down four standard steps (10 inches deep, 6 inches tall) three consecutive times without stopping, and the total time (in seconds) was recorded. ‘Stair climbing’ was calculated as the inverse of the total time recorded (stairs per second). Handrails were available if needed.

#### CPET.

A standardized CPET, using a modified Balke or manual protocol, was administered to participants to measure aerobic fitness (98). Two slow 5-minute walking tests were conducted before and after the maximal effort test to assess walking energetics at a preferred walking speed and a slow fixed speed of 1.5 mph. Participants who were excluded from the maximal effort symptom-limited peak test had acute electrocardiogram (ECG) abnormalities, uncontrolled blood pressure, or a history of myocardial infarction, unstable angina, or angioplasty in the preceding 6 months. Testing continued until the respiratory exchange ratio, ratio between VCO_2_ and VO_2_, was ≥ 1.05 and self-reported Borg Rating of Perceived Exertion was ≥ 17. Blood pressure, pulse oximetry, and ECG were monitored throughout exercise. VO_2_peak was determined in the BREEZESUITE software as the highest 30-second average of VO_2_ (ml/min) achieved. Relative VO_2_ (ml/kg/min) was calculated by normalizing average VO_2_ (ml/min) by body weight (kg).

#### Leg power.

Knee extensor leg power was measured using a Keiser Air 420 exercise machine, primarily on the right leg unless otherwise indicated. Participants with recent strokes, aneurysms, cerebral hemorrhages, or abnormal blood pressure were excluded. Resistance was set at 40%,50%,60%, and 70% of the participant’s 1 repetition maximum leg strength to assess power output.

#### Executive function.

The Trail Making Test, Part B, is a timed test that measures executive function, and in particular set-shifting, by evaluating participants’ abilities to connect a sequence of alternating numbers and letters. A faster time to completion (in seconds) represents better task performance. The Trails B exam was stopped when the participant completed the exam successfully, 5 minutes (300 seconds) elapsed or 5 errors were made. The time taken (seconds) to complete the test was recorded. Performance on this executive measure was calculated as the inverse of this time, so that higher values reflect better task performance.

#### Processing speed.

The Digit Symbol Substitution Test (DSST) from the WAIS-III was used to assess processing speed, motor speed, visuoperceptual functions, attention, and manual dexterity. Scores range from 0–133, with higher scores indicating better cognitive performance.

#### Memory function.

The California Verbal Learning Test (CVLT)-II (Short Form) SF was used to assess verbal learning ability and memory. Participants were read a list of nine nouns (from three categories: fruits, tools, and clothing) and were asked to recall as many as possible after each repetition. The process was repeated four times. After a 10-minute delay, participants were asked to recall the list again. Correct answers and intrusions were noted. Immediate recall was used to measure memory performance, and was calculated as the sum of items correctly recalled across the four learning trials (range 0–36).

#### Contrast sensitivity.

Contrast sensitivity is a fundamental component of visual processing involved in supporting various higher-order cognitive processes. Contrast sensitivity was included as a variable of interest given evidence of age-related contrast deficits, which have been linked to increased risk of cognitive decline, dementia, and mobility limitations. Contrast sensitivity was measured using the Pelli-Robson eye chart. Correct answers were converted to log contrast sensitivity (LCS) scores. Lower LCS values indicate worse contrast sensitivity.

### ASAT biopsy collection and processing

A detailed description of biopsy sample collection and processing is available here (100). Briefly, ASAT biopsies were performed via tumescent liposuction (with a maximum of three needle passes) between the flank and the abdominal midline, below the umbilical scar. ASAT samples were flash-frozen in liquid nitrogen for subsequent bulk RNA sequencing. A portion of ASAT sample was immediately transferred to M199 for conditioned media experiments (see details below).

### Analytical procedures

#### ASAT explant conditioned media.

Transferred ASAT samples were rinsed with warm Hank’s Balanced Salt Solution (HBSS) (ThermoFisher Scientific) to remove visible blood clots and connective tissue. Pre-warmed M199 containing 25 mM Hepes was used at a ratio of 100 mg of ASAT to 2 mL media. ASAT sample was t transferred to a 50 mL conical tube containing media which was gassed then with 95%02 for 30 seconds and tightly sealed. ASAT explants were incubated in a shaking water bath at 37°C and 100 rpm for 3 hours. After incubation, the ASAT and CM were poured through a 40μm cell strainer (Fisher Scientific) into fresh 50 mL conical tubes. The CM was centrifuged at 500 × g at room temperature for 5 minutes, then aliquoted into multiple vials for storage at −80°C at the biorepository.

#### Targeted assessment of secreted proteins.

Enzyme-linked immunosorbent assay (ELISA) was used to measure the abundance of adiponectin and leptin in the CM(N = 168), following the manufacturer’s protocols. The measured abundance was normalized to ASAT explant mass.

#### LC-MS/MS-based proteomics.

Protein from ASAT explant conditioned media samples was isolated and processed for mass spectrometry-based analysis using S-Trap micro sample processing columns (Protifi, Fairport NY) with a protocol adapted from manufacturer recommendations. Briefly, 200 μL CM aliquots were concentrated to 90 μL in a vacuum centrifuge, mixed 1:1 with lysis buffer (10% SDS, 100mM TEAB pH 8.5), reduced with dithiothreitol (DTT, final concentration 20 mM) at 55°C for 10 minutes, and alkylated with iodoacetamide (IAA, final concentration 40 mM) in the dark at room temperature for 30 minutes. Samples were acidified to pH < 1, mixed with binding buffer (100 mM TEAB in 90% methanol), and applied to S-Trap micro columns via centrifugation. Captured proteins were digested with 2.5 μg of sequencing-grade trypsin per sample for 2 hours at 47°C, and digested peptides were eluted from columns according to manufacturer’s protocol. Digested peptides were concentrated in a vacuum centrifuge, measured by BCA assay, and resuspended to a final concentration of 0.1 μg/μL in 3% acetonitrile/0.1% formic acid solution. Peptides (0.5 μg per sample) were separated using a Vanquish Neo UHPCL system (ThermoFisher Scientific) equipped with a homemade 75 μm I.D. × 30 cm length separation column packed with Acquity 130A, 1.7 μm BEH-C18 (Waters) and a homemade 150 μm I.D. × 5 cm length trapping column packed with Jupiter 300A, 5 μm C18 (Phenomenex). A 120-minute gradient of 100% mobile phase A (0.1% formic acid in water) to 75% mobile phase B (0.1% formic acid in acetonitrile) was applied to each sample, and the separation was coupled to an Orbitrap Exploris 240 mass spectrometer (ThermoFisher Scientific) for MS/MS analysis. MS spectra were collected from 300–1800 m/z at an MS1 resolution setting of 60,000. Ions were selected with an isolation width of 0.7 m/z for higher energy collision dissociation (HCD, 30%); +1 charged species were excluded, and a dynamic exclusion window of 45 seconds was applied. MS2 spectra were acquired at a mass resolution of 30,000 and a normalized AGC target of 250%. Raw mass spectrometry data files were analyzed with MaxQuant software (v2.2.0.0) against a human UniProt database (downloaded in March 2023) with the following parameters: LFQ and match-between-run enabled, fixed modifications of cysteine carbamidomethylation, variable modifications of methionine oxidation and n-terminal acetylation, 20 ppm mass tolerance, and a maximum of 4 missed cleavages. Protein-level LFQ intensity data (from the resulting proteinGroups.txt file from MaxQuant analysis) was log2-transformed and median-centered within each sample. Batch correction was applied using limma::removeBatchEffect to account for ten samples that were processed and analyzed in a separate batch, and this dataset (log2-transformed, median-centered, batch-corrected) was exported for use in downstream analysis.

#### ASAT bulk RNA sequencing.

Frozen ASAT samples were homogenized using pellet pestle cordless motor. RNA was subsequently extracted using the RNeasy Lipid Tissue Mini Kit (Qiagen #74804). Nanodrop Spectrophotometer was used to measure RNA concentration and ratio. Library preparation was conducted using Mini Amp Plus Thermal Cycle (Thermo Fisher) and magnetic racks (Zymo Research) with a total of 500 ng of RNA. Library concentration was measured using Qubit dsDNA HS Assay Kit (Invitrogen Q32851) and the Qubit 4 Fluorometer device (Invitrogen). Individual libraries were pooled and sequenced using a NovaSeq 6000 (Novagene) following in-house established protocols which also run quality control checking. Raw RNAseq reads were inspected for quality using FastQC v0.11.9 (Barbraham Institute, Barbraham, England). Reads were then aligned to the human reference transcriptome (Homo_sapiens.GRCh38.cdna v94) using kallisto (101). Count matrix was produced by averaging kallisto TPM at the gene-level per sample.

### Bioinformatics

#### Proteomics.

Among 2366 proteins that were identified, 1276 proteins were detected by at least 25% of participants. Proteins were subsequently filtered for secreted proteins, using the secreted protein list downloaded from The Human Protein Atlas (Supporting information 1), retaining 275 secreted proteins.

Only these 275 secreted proteins were used for downstream analyses. For comparison of secreted proteins between stratified tertiles (Fig. 6C, D), limma R package (version 3.62.1) was used.

#### Weighted gene co-expression network analysis (WGCNA).

WGCNA (102) (version 1.73) was used to identify non-overlapping clusters (modules) of ASAT transcripts. Connectivity for each transcript was determined by summing its connection strengths with all other transcripts. A scale-free signed network was built using an optimal soft-threshold power (β = 5). From this network, 10 distinct modules (including Module 0) were identified using a dynamic tree-cutting algorithm and a merging threshold of 0.3. Module 0, often referred to as the ‘grey’ module, contains transcripts that failed to cluster into other modules due to weak correlation patterns. Top hub genes per module were extracted by their kME (module eigengene-based connectivity). MEs, the principal eigenvector of each module, were extracted and biweight midcorrelation was used to assess the relationship between modules and clinical traits/secreted protein abundances.

#### Gene set analysis.

Overrepresentation analysis (ORA) was conducted using hypeR R package (version 2.4.0). Gene Ontology Biological Process (Fig. 2F, S4C) and HALLMARK (Figure S2C) were used as reference databases. Enrichplot R package was used to conduct rank-based geneset enrichment analysis (Figure S2B). The top 10 terms (FDR < 0.05) for each module were shown (Fig. 2F).

#### Cell-type gene markers.

Cell-type gene markers were identified from ‘Markers for human clusters and subclusters’ obtained from Emont et al., 2022 (29). A threshold of log2FC > 1 was further applied to retain gene markers that are specifically expressed.

#### Senescence gene markers.

A customed senescence geneset (n = 1369) was curated by combining previously reported datasets for cellular senescence: GO term (GO:0090398, cellular senescence), CellAge (31), SenMayo (32), and AgingAtlas (33).

#### Gene Derived Correlation Across Tissues (GD-CAT).

GTEx data (n = 310) was adopted from GD-CAT pipeline (55), with samples filtered by age into younger (n = 175) and older (n = 135) cohorts (56), based on the provided age ranges. Transcriptomics correlations were calculated using biweight midcorrelation (bicor) via the bicorAndPvalue() function in the WGCNA package. Correlation values (bicor) and corresponding p-values were processed and adjusted for multiple testing using the Benjamini-Hochberg (BH) method to control the false discovery rate. For visualizing significant correlations, bar plots were generated for each gene to depict the tissue distribution of significantly correlated genes, stratified by age group. Gene set enrichment analysis (GSEA) was performed downstream using highly correlated genes. Genes were ranked by bicor values, and the gseGO() function from the clusterProfiler R package was used to perform GSEA. The analysis utilized the “org.Hs.eg.db”annotation database for human genes, and pathways were evaluated across Gene Ontology (GO) categories (“Biological Process,”“Molecular Function,”and “Cellular Component”). Adjusted p-values (BH correction) were used to identify significant pathways. Pathway enrichment results were stratified by age and displayed for both positive and negative correlation subsets.

### Sample size calculation and statistics

To calculate the sample size for proteomics, we used a previous study that compared irisin (secreted protein often induced by exercise) levels between non-exercised and exercising individuals (103). The mean level of irisin in a non-exercised group was 3.6ng/ml with an estimated IQR of 3.3–3.9ng/ml. The fold change between exercised and non-exercised groups was 1.2. Therefore, to reliably detect differences in irisin with the aforementioned criteria would require N = 37 subjects. Based on these estimates, for two-sided a of 0.05 and α power of 95%, we determined that 60 samples were needed with the comparison against a non-explant control. Biweight midcorrelation was used for correlation analysis (Figs. 1C, 2 (A, B), 3 (B, C E, F), 4 (A–C), S2A, and S3 (B, D)). For covariate adjustments, residual adjustment approach was used, where clinical traits and protein abundances were separately regressed on the covariates (sex, age, and ASAT volume) using linear regression models. The residuals from these models, representing the variation in clinical traits and protein abundances after accounting for the effects of the covariates, were extracted. Biweight midcorrelation was then performed on the residuals to assess the adjusted association. Spearman’s R correlation was used for non-normalized variables (Figures S2A and S4B). To assess statistical differences between stratified tertiles in females and males (Fig. 6E, F), two-tailed independent Student’s t-tests were used within each group. Benjamini-Hochberg was used to correct for multiple testing. Statistical computations were performed using R (version: 4.4.2, Vienna, Austria). All experiments were performed once.

## Figures and Tables

**Table 1:** Baseline anthropometric characteristics of study participants.

	Overall	Tertile 1	Tertile 2	Tertile 3	
	(N = 229)	(N = 74)	(N = 74)	(N = 74)	P-value (ANOVA)
**Age, y**	76.1 (4.4)	76.7 (4.9)	76.1 (4.1)	75.2 (3.8)	0.109
**Sex**					1.88E-08
F	120 (52.4%)	22 (29.7%)	38 (51.4%)	57 (77.0%)	
M	109 (47.6%)	52 (70.3%)	36 (48.6%)	17 (23.0%)	
**Weight, kg**	78.2 (14.7)	69.7 (11.9)	78.0 (12.4)	86.0 (14.7)	7.57E-12
**BMI, kg/m2**	28.1 (4.3)	24.5 (2.8)	27.9 (2.8)	31.8 (3.5)	< 2e-16
**Waist circumference, cm**	95.9 (13.3)	88.0 (11.5)	96.4 (12.4)	102.4 (115)	6.96E-11
**ASAT, L**	7.84 (3.06)	4.62 (0.99)	7.56 (0.78)	11.35 (1.91)	< 2e-16
**VAT, L**	4.57 (2.21)	3.71 (1.96)	4.74 (2.19)	5.24 (2.21)	7.20E-05
**Liver Fat, %**	4.60 (4.23)	3.38 (2.83)	4.60 (3.78)	6.78 (5.78)	0.00387
**Thigh Muscle Fat Infiltration, %**	7.32 (2.12)	6.65 (2.24)	7.12 (1.78)	8.20 (2.02)	1.87E-05
**D3CR Muscle Mass, kg/weight**	29.2 (6.9)	33.9 (6.9)	28.5 (4.7)	25.6 (6.1)	4.10E-14
**VO2peak, L/min**	1.58 (0.44)	1.68 (0.49)	1.51 (0.39)	1.55 (0.42)	5.50E-02
**VO2peak, ml/kg/min**	20.3 (4.8)	23.9 (5.3)	19.4 (3.7)	17.8 (2.9)	2.34E-16

Data given as mean (SD) except sex; n (%). *P*-values reflect significance for linear trend following one-way ANOVA for differences among ASAT tertiles. Tertile 1, 2.18–6.09 L ASAT; Tertile 2, 6.16–8.97 L ASAT; Tertile 3, 9.01–18.2 L ASAT. SD, Standard Deviation; BMI, Body Mass Index; ASAT, Abdominal Subcutaneous Adipose Tissue; VAT; Visceral Adipose Tissue; MFI, Muscle Fat Infiltration. Subjects without SAT measurements (n = 7) are included in the total, but not in tertiles.

**Table 2:** Participant characteristics of LOW (tertile 1) and HIGH (tertile 3) of “Physical fitness” whose ASATCM was analyzed.

	Female		Male	
	LOW	HIGH	LOW	HIGH
	(N = 9)	(N = 9)	(N = 12)	(N = 12)
**Age, y**	78.8 (5.1)	74.7 (3.6)	78.0 (4.5)	73.3 (2.0)**
**Weight, kg**	72.7 (9.45)	76.0 (14.3)	94.2 (14.3)	82.5 (12.4)*
**BMI, kg/m2**	29.8 (3.9)	29.4 (5.0)*	31.2 (4.0)	26.5 (2.6)**
**Waist circumference, cm**	92.4 (10.3)	90.1 (10.1)*	105 (16.6)	96.4 (13.9)
**ASAT, L**	9.09 (2.08)	9.50 (3.23)	8.27 (2.98)	5.88 (1.84)*
**VAT, L**	3.81 (1.19)	3.75 (1.87)	7.94 (2.19)	4.90 (2.08)**
**Liver Fat, %**	6.38 (4.66)	10.1 (13.1)	5.99 (3.22)	3.14 (1.64)
**Thigh Muscle Fat Infiltration, %**	8.30 (1.82)	6.28 (0.61)	7.17 (1.26)	5.42 (1.00)**
**D3CR Muscle Mass, kg/weight**	26.5 (3.1)	29.4 (3.6)*	29.6 (4.8)	36.6 (5.2)**
**VO2peak, L/min**	1.22 (0.18)	1.54 (0.20)	1.86 (0.31)	2.25 (0.29)**
**VO2peak, ml/kg/min**	16.8 (1.94)	20.6 (3.74)*	19.9 (2.85)	27.5 (3.18)****
**Walking speed (4m), m/s**	0.99(0.17)	1.20 (0.26)**	1.05 (0.09)	1.22 (0.10)***
**Stair climbing, stairs/s**	1.40 (0.61)	1.78 (0.43)**	1.53 (0.17)	1.91 (0.25)***
**Leg power (1RM), Watt/kg**	2.79 (1.20)	5.03 (1.08)***	4.69 (1.36)	7.56 (0.91)****
**Executive function** ^#^ **, sec**	130.0 (82.3)	93.3 (28.5)	120.0 (51.1)	80.8 (22.1)*
**Processing speed, score**	49.7 (20.8)	58.4 (16.3)***	53.3 (9.7)	59.1 (8.3)
**Memory, score**	24.0 (4.8)	27.2 (2.4)	23.3 (4.5)	27.6 (5.1)
**Contrast sensitivity, score**	1.47 (0.14)	1.59 (0.11)**	1.55 (0.15)	1.63 (0.15)

* p < 0.05

** p < 0.01

*** p < 0.001

**** p < 0.0001 within sex group.

# Executive function is shown in original values (time to complete all tasks), thus higher value indicates slower speed.

## Data Availability

All datasets used in this manuscript are available at (https://sommaonline.ucsf.edu/). Data as of November 2024 were used in this study.
